# An In Vitro Human Skin Test for Predicting Skin Sensitization and Adverse Immune Reactions to Biologics

**DOI:** 10.3390/toxics12060401

**Published:** 2024-05-30

**Authors:** Shaheda Sameena Ahmed, Mohammed Mahid Ahmed, Abbas Ishaq, Matthew Freer, Richard Stebbings, Anne Mary Dickinson

**Affiliations:** 1 Alcyomics Ltd., The Biosphere, Draymans Way, Newcastle Helix, Newcastle Upon Tyne NE4 5BX, UK; shaheda.ahmed@hotmail.com (S.S.A.); mahid.ahmed9@gmail.com (M.M.A.); abbas.ishaq@alcyomics.com (A.I.); matthew.freer@alcyomics.com (M.F.); 2 National Institute for Biological Standards and Control, Blanche Lane, South Mimms, Potters Bar, Hertfordshire EN6 3QG, UK; richard.stebbings@astrazeneca.com; 3 Translational and Clinical Research Institute Faculty of Medical Sciences, Newcastle University, Newcastle-upon-Tyne NE2 4HH, UK

**Keywords:** skin explant, adverse event, T cell proliferation, monoclonal antibodies, cytokine release

## Abstract

Biologics, including monoclonal antibodies (mAb), have proved to be effective and successful therapeutic agents, particularly in the treatment of cancer and immune-inflammatory conditions, as well as allergies and infections. However, their use carries an inherent risk of an immune-mediated adverse drug reaction. In this study, we describe the use of a novel pre-clinical human in vitro skin explant test for predicting skin sensitization and adverse immune reactions. The skin explant test was used to investigate the effects of therapeutic antibodies, which are known to cause a limited reaction in a small number of patients or more severe reactions. Material and Methods: Immune responses were determined by T cell proliferation and multiplex cytokine analysis, as well as histopathological analysis of skin damage (grades I–IV in increasing severity), predicting a negative (grade I) or positive (grade ≥ II) response for an adverse skin sensitization effect. Results: T cell proliferation responses were significantly increased in the positive group (*p* < 0.004). Multiplex cytokine analysis showed significantly increased levels of IFNγ, TNFα, IL-10, IL-12, IL-13, IL-1β, and IL-4 in the positive response group compared with the negative response group (*p* < 0.0001, *p* < 0.0001, *p* < 0.002, *p* < 0.01, *p* < 0.04, *p* < 0.006, and *p* < 0.004, respectively). Conclusions: Overall, the skin explant test correctly predicted the clinical outcome of 13 out of 16 therapeutic monoclonal antibodies with a correlation coefficient of 0.770 (*p* = 0.0001). This assay therefore provides a valuable pre-clinical test for predicting adverse immune reactions, including T cell proliferation and cytokine release, both associated with skin sensitization to monoclonal antibodies.

## 1. Introduction

Biologics, including monoclonal antibodies (mAbs), are widely used as therapeutic agents for the treatment of disease. The development of therapeutic antibodies has advanced over the last few decades. The transition from mouse monoclonal antibodies [1] to humanized forms [2,3] has resulted in reduced immunotoxicity and improved safety and efficacy. However, a low number of adverse events still occur, halting clinical trials, despite the improvement in the use of humanized monoclonal antibodies. Common adverse events include, but are not limited to, skin sensitization, infusion reactions, anaphylaxis, rash, anti-drug antibody (ADA) development, and cytokine release syndrome, as well as other complications [4].

Hypersensitivity involves cytokine release and symptoms, which can range from skin sensitization and rashes to anaphylaxis and organ failure and are classified from type I to IV [5]. Type I immediate adverse response is associated with IgE antibody release and anaphylaxis [6]. A type II response is an antibody-dependent cytotoxic response [7], and a type IV response is a delayed T cell-mediated hypersensitivity response [8]. Marketed monoclonal antibody therapeutics have been associated with hypersensitivity reactions [9,10] involving elevated levels of circulating cytokines (Cytokine Release Syndrome (CRS)) and subsequent serious adverse reactions, including anaphylaxis [11].

Current practices for preclinical safety evaluation of mAbs or biopharmaceuticals involve the use of in vivo animal studies, followed by standard in vitro toxicity studies [12]. The main aim of these pre-clinical studies is to determine the immunotoxic properties of mAbs and determine a safe first-in-human dose to conduct Phase I clinical trials. However, the success of the use of these methods is limited to the selection of relevant animal species and the correct interpretation of animal study data for humans [13,14]. However, this approach can lead to misleading data, as in the case of TeGenero in 2006 [15]. In this trial, cynomolgus and rhesus monkeys were chosen to perform preclinical in vivo studies because of the similarity in their CD28 receptor to the human CD28 receptor and their binding affinity to the TGN1412 antibody [16]. A repeat dose study was performed, and no adverse reactions or toxicity were observed. The immunotoxicity of the TGN1412 antibody, however, did not become apparent until administration to Phase I volunteers, resulting in severe cytokine release syndrome (CRS) and organ failure. There is a pressing need to develop preclinical human in vitro tests that can predict the immunotoxic properties of biologics. To this end, we have developed a novel human in vitro skin explant test to predict cytokine-associated adverse responses, skin sensitization, or immunotoxic properties of biologics as a first-in-man test. The test has been used to predict adverse immune reactions to sensitizers and non-sensitizers [17] as well as hypersensitivity responses to low molecular weight (LMW) drugs [18]. In the current study, the skin explant test was further modified for the safety assessment of biologics. The test mimics the in vivo immunological response upon exposure to a mAb, with the elicitation of an immune response in the form of histopathological skin damage (skin sensitization reactions). The predictive endpoint of the test is based on the observed histopathological tissue damage, which is scored (grades I–IV) in order of increasing severity of damage in the skin [19]. Furthermore, the grading of damage in the skin allows for the determination of dose response. This novel endpoint has not been previously described for any tests predicting cytokine-associated adverse events or direct skin sensitization.

In this study, we investigated the effects of 16 known therapeutic mAbs, each with the potential to stimulate a different arm of the immune system (Fab or Fc).

## 2. Materials and Methods

### 2.1. Healthy Volunteers

This study involved the use of blood (60 mL) and skin biopsies (two 4 mm punch biopsies) taken from 10 healthy volunteers with informed consent at a dermatology clinic by a National Health Service research nurse who also conducted a questionnaire to ensure the volunteers had no underlying condition and were fit and healthy. Each set of blood and skin biopsies was used to test each mAb. This research was approved by the Local Research Ethics Committee (LREC).

### 2.2. Antibodies

Sixteen test antibodies were provided by the National Institute of Biological Standards and Control (NIBSC). Antibodies were selected based on the known ability of the antibody, as defined by the box warnings, to give rise to responses ranging from extreme to weak in the form of a subcutaneous rash or a systemic response causing organ toxicity (Table 1). These included 11 antibodies of the IgG1 subclass: campath-1H (alemtuzumab), mabthera (rituximab), remicade (infliximab), remsima (infliximab), inflectra (infliximab), embrel (etanercept), humira (adalimumab), simulect (basliximab), erbitux (cetuximab), arzerra (ofatumumab), simponi (golimumab), 2 antibodies of the IgG2 subclass orthoclon-OKT3 (muromonab) and vectibix (panitumumab), and 2 antibodies of the IgG4 subclass: tysabri (natalizumab) and a TGN1412 homolog. Finally, the biological PEGylated fragment of the monoclonal antibody cimzia (certolizumab) was also tested. All antibodies were tested at 1 µg/mL. This dose was recommended by colleagues at the NIBSC and was also shown to be within the optimal range in assays for assessment of monoclonal antibodies as published by Stebbings et al. [20], but it was also the minimum dose in pilot studies that produced negative skin explant grades (Grades 0–I) with the weak positive control antibody tysabri and positive grades II–III with the positive antibody OKT3. Human isotype controls were used in all assays; this included IgG1 (A50183H, AMS Biotechnology, Abingdon, UK) for 11 biologics, IgG2 (HCA193, Bio-Rad, formerly AbD Serotec, Hercules, CA, USA) for panitumumab and muromonab, and finally, IgG4 (A01241H, AMS Biotechnology) for natalizumab and TGN1412.

### 2.3. Skin Explant Test

**Figure 1:**
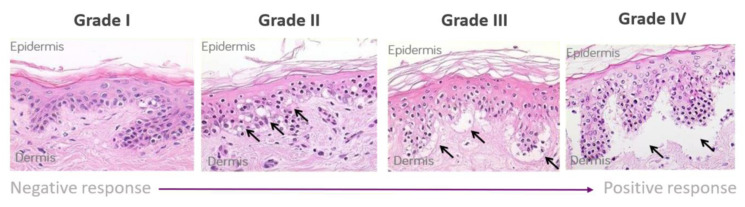
Lerner grading scheme for sub-epidermal lesions in the skin explant assay. The Grade II arrows indicate epidermal skin damage with vascularization and dyskeratosis; the Grade III arrows show Grade II damage as well as sub-epidermal cleft formation; Grade IV arrows show extensive sub-epidermal damage with separation of the dermis and epidermis.

The skin explant tests were performed on a minimum of 10 healthy volunteers. Whole blood (60 mL) was used to isolate peripheral blood mononuclear cells (PBMCs) using the Lymphoprep™ (Gibco, Grand Island, NY, USA) method and density gradient centrifugation. Five ml of whole blood was used to collect serum, which was heat inactivated (56 °C for 30 min) prior to use. Two 4 mm skin biopsies were collected in X-Vivo-10 medium (Lonza, Basel, Switzerland) and were cut into 12 equal sections prior to use. The antibody of interest was co-cultured at 1 µg/mL concentration with or without PBMCs (1 × 10^6^) and autologous skin sections in RPMI 1640 (Gibco, London, UK) containing 100 IU/mL penicillin, 100 µg/mL streptomycin (Gibco UK), and 2 mM L-glutamine (Gibco UK) supplemented with 20% heat-inactivated autologous serum for 3 days. Routinely, all PBMC showed greater than 95% viability with the addition of PBMC to the assay. Background control skin was cultured with medium alone [21]. To determine non-specific binding, the antibody isotype was used as a negative control to assess background histopathological damage. Following the 3-day incubation, supernatants were collected for cytokine analysis, and the skin was paraffin embedded, sectioned, and stained with hematoxylin and eosin. Skin explants were examined blindly by two independent observers for histopathological damage. Using the Lerner histological grading system [19], the damage observed was assigned a grade (I–IV) relative to the severity of the damage observed (Figure 1). The grading system was as previously described [21] and as follows: a grade I response is considered a negative reaction with normal skin pathology but may show some mild vacuolization of cells. Grades II–IV are considered positive reactions. Grade II is like a skin sensitization rash and shows damage in the form of vacuolization of keratinocytes and dyskeratotic bodies. Grade III is analogous to blistering and damage at the dermal/epidermal junction with cleft formation. Grade IV is similar to skin peeling and displays complete separation of the dermis from the epidermis.

### 2.4. [3H]-Thymidine T Cell Proliferation Measurement

T cell proliferation responses were measured in the same donors for the skin explant tests following exposure of PBMC’s to the biologics at 1 μg/mL concentration. T cell responses were correlated to a negative response (grade I) or a positive response (≥grade II) in the skin explant tests for each donor (*n* = 10) and each test antibody. PBMC’s were incubated with the test antibody isotype or control antibody (1 µg/mL) for 5 days in triplicate in RPMI 1640 (Gibco, UK) containing 100 IU/mL penicillin, 100 µg/mL streptomycin (Gibco UK), and 2 mM L-glutamine (Gibco UK) supplemented with 10% heat-inactivated fetal calf serum. Cells were harvested and [3H] Thymidine added for the last 16–18 h. [3H]-thymidine primary stock was stored at 37 MBq and used at 3.7 MBq (1/10 dilution), and subsequent uptake was measured using a microbeta-scintillation counter in counts per minute (cpm). Data were interpreted using Prism GraphPad software (V5, San Diego, CA, USA).

### 2.5. Analysis of Cytokine Concentration in Cell Culture Supernatants

Supernatants collected from the skin explant cultures were measured according to the manufacturer’s instructions by Meso Scale Discovery (MSD) V-PLEX Proinflammatory Panel 1 for human IFNγ, TNFα, IL-10, IL-12, IL-13, IL-1β, IL-2, IL-4, and IL-6.

### 2.6. Statistical Analysis

(1) Skin Explant Data

Skin explant results were assigned grading scores for histopathological damage, and this positive or negative score was then compared with clinical responses and box warnings as reported for each test antibody. The classification of the data was as follows, with the clinical comparison given in brackets. An antibody was a strong positive if 71% or more of the total tests performed gave a grade ≥II response (frequent > 10%), a positive if 31–70% of the total tests performed gave a ≥grade II (common 1–10%), and a weak positive if 10–30% of the total tests performed gave a ≥grade II (uncommon event 0.1–1%). Pearson’s correlation coefficient was used to correlate skin explant grades with clinical outcome data, i.e., frequent, common, uncommon, or rare clinical events. Additionally, average grades over 10 donors for each tested compound were calculated. An aggregate score was then derived by multiplying the average grades by the percentage of positive donors. The classifications and aggregate scores are in-set predictions. The aggregate score was then compared against the clinical frequency classification above using Pearson’s correlation.

Where relevant, *p*-values were adjusted for the false discovery rate using a Benjamini-Hochberg correction. The adjusted values are indicated on the individual graphs.

(2) T Cell Proliferation Responses

T cell proliferation data was obtained by determining the mean cpm from triplicate culture wells of [3H] thymidine incorporation, converted to a log-fold increase to allow for equal distribution of data. T cell proliferation stimulation indices were matched to the graded response observed in the skin explant tests for each donor. Responses were grouped as negative (grade I) or positive (grade ≥ II). T cell proliferation responses were compared between the two groups, and statistical analysis was performed with an unpaired *t* test.

(3) Cytokine Levels in Cell Culture Supernatants

Cytokine levels in cell culture supernatants were measured as concentrations of pg/mL. Cytokine levels were compared between the two groups, and statistical analysis was performed with an unpaired *t* test. A *p*-value of <0.05 was statistically significant. Correlation coefficients were determined using Statistical Package for Social Sciences (SPSS) Statistics 7. Unpaired *t*-tests were replicated in R (R Studio Build 402), and false discovery rate correction was applied using the Benjamini-Hochberg method. The q-values did not significantly modify the *t*-test *p*-values.

## 3. Results

### 3.1. Skin Explant Test Demonstrates a Positive Correlation with Clinical Responses in 14/16 Therapeutic Antibodies

**Figure 2:**
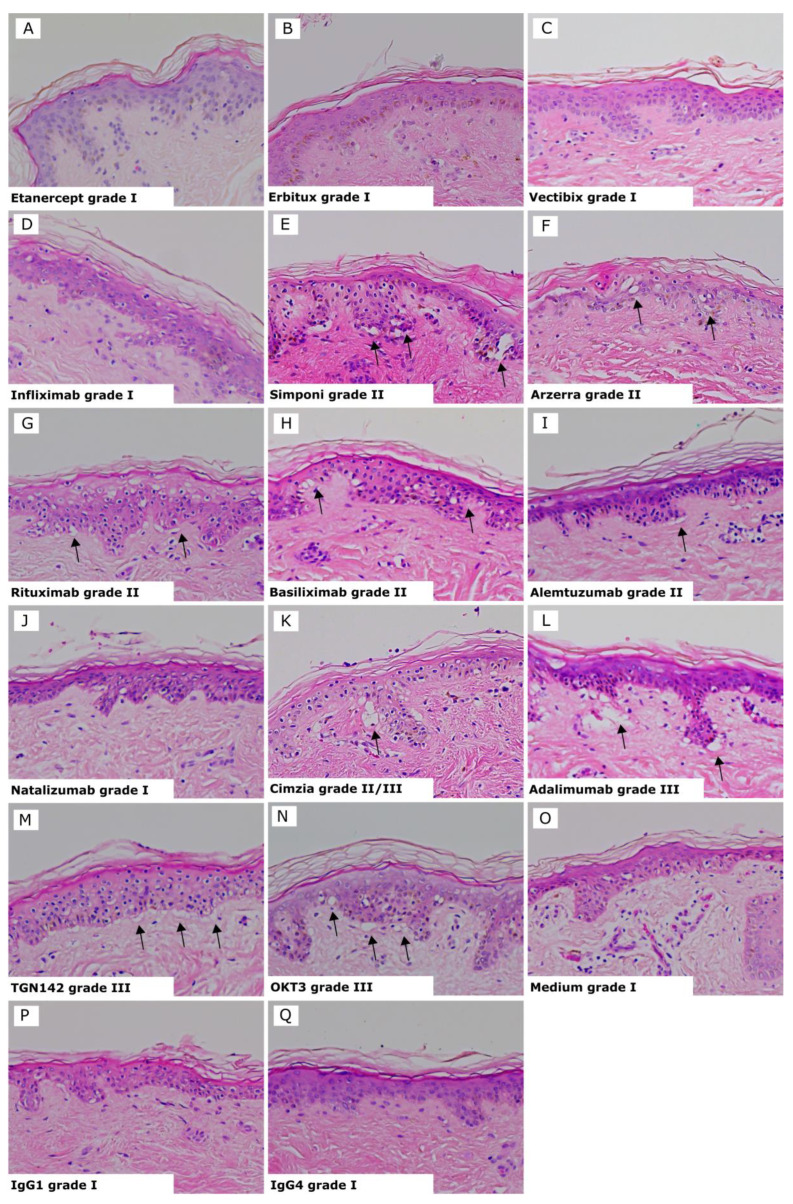
Skin explant results gave either a negative response (grade I) or positive response indicated by black arrows (grades II–III). (**A**) Etanercept grade I; (**B**) Erbitux grade I; (**C**) Vectibix grade I; (**D**) Infliximab, all brands, grade I; (**E**) Simponi grade II; (**F**) Arzerra grade II; (**G**) Rituximab grade II; (**H**) Basiliximab grade II; (**I**) Alemtuzumab grade II with vacuolarization and dyskeratosis of the epidermis; (**J**) Natalizumab grade I; (**K**) Cimzia grade II/III; (**L**) Adalimumab grade III; (**M**) TGN1412 grade III with separation of the dermis and epidermis; (**N**) OKT3 grade III; with vaculorization and dyskeratosis of the epidermis; (**O**) Media grade I; (**P**) IgG1 isotype control grade I; (**Q**) IgG4 isotype control grade I.

Results for each test biologic are summarized in Table 1, and representative images of the biologics showing positive and negative responses and controls are shown in Figure 2. The three Infliximab antibodies are represented as one image, as they all showed the same average grades. Positive reactions presented histopathological damage in the skin consisting of vacuolization of basal cells with some dyskeratotic bodies (grade II) (Figure 2E–I) or sub-epidermal cleft formation (grade III) (Figure 2K–N). Negative reactions showed a grade I response in the skin (Figure 2A–D and J). Isotype antibodies or skin cultured in medium alone were used as negative controls and gave a negative response (grade I) in all tests (Figure 2O–Q).

13 out of the total 16 tested monoclonal antibodies were correctly categorized by the skin explant test according to the box warnings and clinically relevant classifications (frequent, common, uncommon, and rare) observed in clinical trials for immunological reactivity (Table 1). The skin explant categorization was an in-set prediction.

Biologics that were correctly predicted positive were TGN1412 homolog (9 of 10 tests showed a grade ≥ II positive response), OKT3 was positive in 9 of 10 initial experiments and was then subsequently used as a positive control in all tests of the therapeutic antibodies. OKT3 was positive in 29 of 30 tests with a grade >II response. Alemtuzumab showed a grade ≥ II positive response in 7 of 10 tests. Rituximab, cetuximab, ofatumumab, and bevacizumab also showed a positive response (5 of 10 tests were positive, respectively), and Simponi (4 of 10 tests showed a ≥grade II). Panitumumab, infliximab, and certolizumab (3 of 10 tests positive) were also categorized as positive; however, the grading was generally weaker.

Natalizumab (8 of 10 tests showed a grade I negative response) and etanercept (9 of 10 tests showed a grade I negative response) were categorized as weakly positive.

The three incorrectly categorized compounds were simulect and panitumumab, which were overclassified, and etanercept, which was under classified, respectively.

A strong Pearson’s correlation coefficient of r = 0.834 and a *p* value of <0.0001 were observed between the two comparatives (skin explant and clinical outcome data). Furthermore, the percentage of donors positive for a skin explant test reaction strongly correlated with the average grade across all 10 donors (r = 0.896).

### 3.2. T Cell and IGNɣ Responses Correlated with Responses Induced by Therapeutic Biologics and Histopathological Damage Observed in Skin Explants

**Figure 3:**
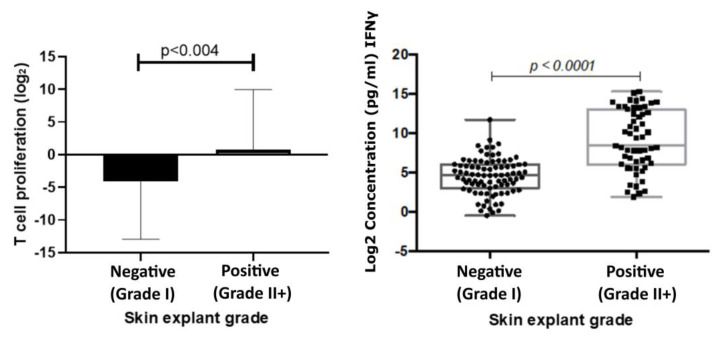
T cell proliferation responses (**left**) correlated with responses observed in skin explant tests. T cell proliferation responses were measured following incubation of PBMCs with test biologics. The graph shows a comparison of Log2 stimulation indices of T cell proliferation, calculated from the base line T cell proliferation responses, to responses observed in the skin explant tests. Data show a significant increase (*p* < 0.004) in T cell proliferation in the positive (grade >II; square symbols) skin explant group compared with the negative (grade I; round symbols) skin explant group. IFNγ responses (**right**) were measured in cell culture supernatants from the skin explant tests. The graph shows Log2 IFNγ levels (pg/mL). IFNγ responses showed a significant increase (*p* < 0.0001) in the positive skin explant group (grade ≥ II) compared with the negative skin explant group (grade I). The *p*-values are FDR-adjusted after a Benjamini-Hochberg correction.

The mean T cell proliferation observed in the positive group was 0.79 ((Log_2_) ± 1.28 SEM) and in the negative group −4.15 ((Log_2_) ± 1.23 SEM). A significant increase in T cell proliferation was observed in the positive response group (*p* < 0.004) compared with the negative response group. Results showed a correlation coefficient of 0.61 (*p* < 0.0001) with IFNɣ levels and skin explant grades (Figure 3).

### 3.3. Cytokines were Increased in Cell Culture Supernatants from Positive Skin Explant Tests

**Figure 4:**
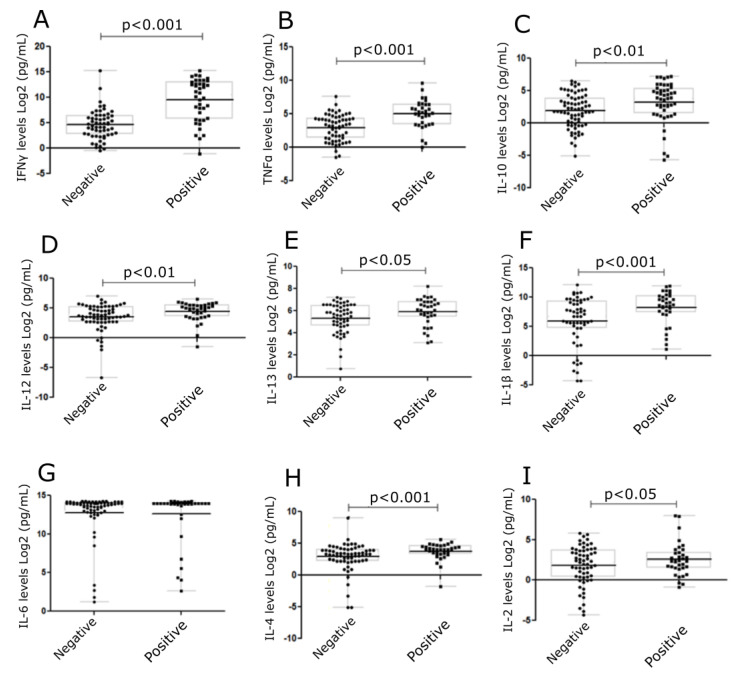
Levels of cytokines in skin explant cell culture supernatants following exposure to mAbs. Cytokine levels were determined in the skin explant test cell culture supernatants. Graphs show cytokine levels (Log2 pg/mL). A significant increase was observed for (**A**) IFNγ (*p* < 0.0001), (**B**) TNFα (*p* < 0.0001), (**C**) IL-10 (*p* < 0.01), (**D**) IL-12 (*p* < 0.01), (**E**) IL-13 (*p* < 0.05), (**F**) IL-1β (*p* < 0.001) (**G**) IL-6, (**H**) IL-4 (*p* < 0.001), and (**I**) IL-2 (*p* < 0.05). The *p*-values are FDR-adjusted after Benjamini-Hochberg correction. The square symbols represent the positive group and the round symbols represent the negative group.

Cytokines, IFNγ, TNFα, IL-10, IL-12, IL-13, IL-1β, IL-6, IL-4, and IL-2 were measured (Figure 4) in cell culture supernatants collected from skin explant assays. Cytokine levels were correlated to negative (grade I) or positive (grade ≥ II) responses in the skin explant tests. Significantly increased levels of IFNγ, TNFα, IL-10, IL-12, IL-13, IL-1β, and IL-4 were observed in the positive response group compared with the negative response (*p* < 0.0001, *p* < 0.0001, *p* < 0.002, *p* < 0.01, *p* < 0.04, *p* < 0.006, and *p* < 0.004, respectively). No differences were observed in IL-2 and IL-6 levels between the positive and negative groups. In the later case, this was due to increased IL-6 levels being produced in normal skin cultures in medium alone (Figure 4).

### 3.4. TGN1412 Showed a Delayed Time Response In Vitro

**Figure 5:**
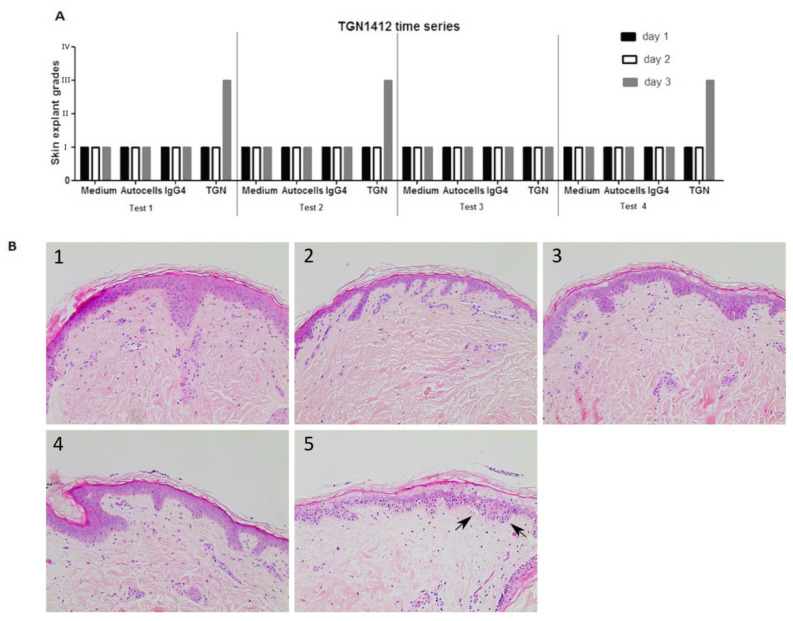
(**A**) In vitro responses (*n* = 4) of TGN1412 over a 3-day time period, day 1 (black bar), day 2 (white bar), and day 3 (grey bar). (**B**) Skin incubated in medium control or with autologous cells or an IgG4 isotype antibody gave a grade I negative response in the skin explant tests on days 1, 2, and 3. TGN1412 gave a grade I negative response on days 1 and 2. On day 3, a grade III positive response was observed in 2/3 tests (as shown by the arrows) and a grade I negative response in the day 1 test. (**B**) Representative images of (1) medium day 3, (2) autologous cells day 3, (3) IgG4 day 3, (4) TGN1412 day 1, (5) TGN1412 a day 3.

TGN1412 was co-cultured with healthy skin and autologous PBMC over a period of 3 days. A skin sample was removed every 24 h, and histopathological analysis was performed. Data (*n* = 4) clearly showed TGN1412 exposure did not cause any damage to skin until day 3 following antibody co-incubation with skin and autologous cells. Negative controls (medium alone incubated with skin, autologous T cells incubated with skin, or the IgG4 isotype antibody incubated with skin in the absence of autologous cells) showed a grade I negative response over the 3 days. TGN1412 exerted a significant immune response, showing an increase in histopathological damage from a grade I negative response at 24 h to a grade III positive response by 72 h (Figure 5), in 2/3 tests, indicating a time-dependent response relationship.

## 4. Discussion

Adverse reactions in response to biological drugs can present in several ways. To further complicate this matter, the true incidence of occurrence is largely unknown. Reaction incidences are reported as variable, influenced by drug dosage, multiple drug administrations [22,23], and even geographical location [24]. Additionally, studies are often reported in small patient groups. In vitro tests such as patch tests, skin prick tests, and intradermal tests are sometimes used; however, some patients still present with adverse immune reactions, including skin sensitization, despite obtaining a negative result in in vitro diagnostic tests [25,26,27]. Steroid drug administration is a frequently used strategy to dampen adverse effects of drugs; for example, adalimumab has been shown to cause an adverse reaction in up to 12% of rheumatoid arthritis (RA) patients [28] when administered alone, and this has been seen to be reduced to 1% when co-administered with methotrexate [29,30].

During the TeGenero (TGN1412) clinical trial, the adverse events in response to TGN1412 started to present as early as 3 h post-infusion, with multiple organ failure occurring by 12 h [21]. We used the skin explant assay to determine if the response period of the immune system in vitro was similar to that observed in vivo. The TGN1412 homolog antibody was tested at 1 μg/mL, the estimated concentration to be present in the blood of individuals during the Northwick Park trial [22]. An interesting skin explant assay result was the delay in the response in vitro to the TGN1412 mAb in comparison to the early response reported in vivo. This delayed response may well not be specific to that of TGN1412 and may be related to the fact that autologous PBMCs, which are immature, were used and required a period following activation to become effectors and cause damage.

Following the TGN1412 Northwick Park trial, the need and usefulness of human in vitro studies to determine human safety data and dose ranges have become increasingly recognized. This is illustrated by the work of Stebbings et al. (2007) [20], where they compared several protocols to assess cytokine release, with the most successful being when TGN1412 was immobilized on endothelial cells and demonstrated that the simple interaction of TGN1412 with peripheral blood lymphocytes was not sufficient to cause cytokine release as shown in vivo. The need for improved in vitro assays to predict adverse immune reactions to biologics occurring in vivo has also been debated for some time [31]. This was recently highlighted last year by the FDA in their new Modernization Act to reduce reliance on animal model systems and develop new ways to de-risk novel therapeutics. The key aim of our study was to develop and evaluate a suitable pre-clinical human in vitro test that could predict cytokine release associated with immunotoxicity and other immunological reactions in humans as a first-in-man test to bridge the gap between animal studies and human studies and to avoid a similar occurrence as observed in the TeGenero trial, or skin sensitization or injection site reactions that could halt a clinical trial.

We have shown that the human in vitro skin explant test is able to detect an unwanted immune response to a therapeutic monoclonal antibody. By using an autologous PBMC population and co-culturing these with autologous skin in the presence of the test biologics, we observed histopathological damage in the skin if the therapeutic had caused an adverse immune response in the clinic. As a result, the test distinguished between extreme positive or limited adverse responses to mAbs. In conjunction with other in vitro tests, this test could prove to be valuable during both the early and preclinical development stages of mAbs. It can offer the opportunity to further test modified compounds, for example, protein modification, to decrease any immunotoxic potential and enable compound elimination early in the drug development pipeline.

The skin explant test is dissimilar to alternative tests available for immunotoxicity testing in that it offers observation of histological changes in the skin tissue and observation of skin sensitization as a direct result of immune cell activation and cytokine release. The use of autologous immune cells in this model allows the model to closely mimic an in vivo immune response in vitro.

Cytokine release assays are recognized as a valuable alternative to determining a risk-based evaluation [32] and as early indicators of cytokine release syndrome. Cytokine release observed in the positive Mab skin explants corresponded to Th1 (IFNγ and TNFα) and Th2 (IL-13, IL-10, and IL-4) type responses. Our results were able to replicate previous reports of increased IFNγ [20] or TNFα [31] levels, but not IL-2. T cell proliferation upon exposure to positive mAbs gave positive responses, as previously reported [30].

In an attempt to develop safer biologics, predictive modeling has attracted much interest as a tool for the identification of potential health hazards. A large number of these models are based primarily on the use of drug molecular structure and its activity in a quantifiable approach known as quantitative structure-activity relationship (QSAR) models [33]. They do, however, have limitations. [34]. Another approach involves the development of algorithms allowing T cell epitope mapping to predict or identify immunotoxic epitopes [35,36]. In comparison to in vivo studies, which are time-consuming and expensive, the speed and low cost of these protein immunotoxicology tools make them very attractive. However, they do not provide any information with regard to T cell activation or the prediction of cytokine release.

Recent efforts have also combined the use of in silico and in vitro prediction tools to develop better models to predict adverse reactions, such as the Epibase^®^ in silico and in vitro tools. In vivo animal studies also face much criticism for the pre-clinical assessment of drugs, as animal models can often fail to predict the human immune response, as highlighted by the 2006 TGN1412 clinical trial [37]. There are regulatory and ethical issues around the use of animals in pre-clinical research, as well as the interpretation of the cross-species barrier, and the effectiveness of these models is an important topic of debate [38].

The skin explant test, which effectively distinguishes between monoclonal antibodies showing extreme positive or limited responses, could be used as a valuable preclinical tool in the safety assessment of biologics.

Three compounds were incorrectly categorized, namely simulect and panitumumab, overestimated and etanercept underestimated. Panitumumab carries a box warning for dermatological toxicities and induces a severe skin reaction in approximately 16% of patients. It is therefore likely that the misclassification of panitumumab from uncommon (clinical) to common (skin explant test) was due to the skin-related pathology rather than predicting a more generally immune-mediated reaction. Similarly, Simulect carries a box warning for skin ulceration [39].

Skin-specific reactions are not uncommon for the majority of the biologics tested in this study, including injection site reactions, rash, and inflammation, which are commonly observed [39].

In an exploratory approach to further stratify the skin explant test classification (Table 1), the grading over 10 donors was averaged, using 1–4 rather than the I–IV classification. The grades were grouped as <1.2 for negative reactions, 1.3–2.0 for weak positives, and 2.0–3.0 for strong positives. The percentages were grouped as <10% for negative reactions, 10–30 for weak positives, 31–70 for positives, and 71–100 for extreme reactions. Hypothetically, the product of percentage positive donors and average grade would produce an aggregated score that is more comprehensive than either parameter alone. Due to the strong correlation between both groups (r = 0.770), the aggregated score strongly reflects the percentage group. However, the percentage group parameter does not account for edge cases where 1 or 2 out of 10 donors may be grade 3, or 10 out of 10 donors may be grade 2, whereas the aggregated score rates the former as 1.5 and the latter as a 2. The aggregate score would represent severity and occurrence, where high occurrences of low grades are as noteworthy as low occurrences of high grades. The classifications were derived in-set, and the strong Pearson’s correlation between the various parameters could partially be due to overfitting of the predictions. Further ongoing testing of the skin explant test assay using a targeted selection of novel therapeutics, graded by blinded observers and correlated against clinical trial data, has indicated that the skin explant test assay is predictive. The classification and correlation analysis performed for the 16 therapeutic antibodies analyzed in this study will be repeated with other therapeutics in on-going studies.

Another point to consider is that testing was performed in a healthy donor population, and responses in clinical patients may differ. As a large proportion of monoclonal antibodies are targeted towards immune-related disorders, patients with these disorders may have altered or enhanced immune responses. This could be one reason why commonly positive biologics such as etanercept were classified as weak positives in this test.

As of 2023, the FDA no longer requires animal testing for new therapeutics. It is anticipated that ex vivo human disease models, instead of murine models, will gradually be seen as more relevant models of human diseases. The skin explant assay is a patented technology (traded as Skimune^®^), and additional validation data is ongoing through the testing of blinded compounds from industry where clinical outcomes are known. As demonstrated, the classification criteria are strongly correlated to the histopathology grades. Thus, for underpowered studies with fewer than 10 donors, the average grades are a good indicator of a positive or negative response in the assay and can be used as a pre-clinical screen with the understanding that no mAb is completely negative and that a very small population may show weak positivity with regard to adverse immune events. This, however, does not preclude them from being marketable products.

Further work is being performed to stratify the skin explant assay grading between subepidermal damage and epidermal damage, such as toxic epidermal necrolysis [40,41], and to include acantholysis as demonstrated in palmar-plantar erythrodysesthesia.

## 5. Conclusions

Overall, the results suggest that the skin explant test could be an effective tool in bridging the gap between animal studies and first-in-human clinical trials. The skin explant assay involves the skin sensitization pathway, a molecular initiating event, an inflammatory response and release of cytokines, and an adverse event in the form of keratinocyte activation culminating in skin damage. The assay, therefore, significantly mimics the adverse event pathway of skin sensitization. This assay accurately predicts the adverse reaction incidence rate for a variety of biological therapeutics with varying mechanisms of action and provides a valuable and ethical alternative to animal studies for immunological activity evaluation. The test could also reduce the time and cost of the drug development process by identifying hazardous biological candidates likely to fail in Phase I clinical trials and identifying early skin sensitization reactions implicated in their failure.

## 6. Patents

Alcyomics holds patents on the skin explant assay (trading as Skimune^®^). See the table below.
**Case Code****Applicant****Country****Status****Official Number****Title**P131663CHAlcyomics LimitedSwitzerlandGranted‘EP2524227Skin Explant AssayP131663DEAlcyomics LimitedGermanyGranted‘602011004834Skin Explant AssayP131663DKAlcyomics LimitedDenmarkGranted‘EP2524227Skin Explant AssayP131663ESAlcyomics LimitedSpainGranted‘EP2524227Skin Explant AssayP131663FRAlcyomics LimitedFranceGranted‘EP2524227Skin Explant AssayP131663GB1Alcyomics LimitedUnited KingdomGranted‘EP2524227Skin Explant AssayP131663ITAlcyomics LimitedItalyGranted‘502014902254005Skin Explant AssayP131663USAlcyomics LimitedUSAGranted‘9,651,544Skin Explant AssayP131663USD1Alcyomics LimitedUSAGranted‘10,073,084Skin Explant Assay

## Figures and Tables

**Table 1:** Therapeutic antibodies tested in the study. Antibodies were selected for their known clinical outcome for adverse events. The antibody name, type, target, mechanism, clinical class of response, positive (extreme, strong, or weak) or negative, box warnings occurrence is given. The total number of tests that gave a positive histopathological response (grade II and above) over the total number of tests performed (*n* = 10) is shown and expressed as a percentage, and the average skin explant grade (converted to grades 1–4) (rather than expressed as roman numerals) from the results of the ten donors is also given. A √ tick indicates a correct correlation with expected clinical responses. Responses are shown as weak positives 10–30%, positives 31–70%, and extreme responses >71%.

Antibodies	Target	Mechanism of Action (MOA)	Box Warning	Clinical Classification	Most Common Adverse Reaction	% Positive Skin Explant Test	Skin Explant Test Classification	Percentage Group	Average Skin Explant Test Grade (10 Donors)	Aggregated Score (Grade * Percentage)
OKT3 (Muromonab)	CD3	Blocks T cell activation	Anaphylaxis, Cytokine storm	Extreme (>10%)	Cytokine release syndrome, Pyrexia	96% (√)	Strongly Positive	71–100	2.8	2.7
NIB1412 (TGN1412)	CD28	Activation of regulatory T cells	Caused severe systemic immune reaction	Extreme (>10%)	Cytokine storm	90% (√)	Strongly Positive	71–100	2.3	2.1
Campath^®^ (Alemtuzumab)	CD52	Binds CD52 protein on lymphocytes	Systemic immunogenicity, Rash, urticaria, erythema.	Common (6%)	Infusion reactions, fever	70% (√)	Positive	71–100	2.4	1.6
Humira^®^ (Adalimumab)*	TNFα	Prevents TNF receptor activation, down regulates inflammation	Skin reactivity and hypersensitivity	Common (7%)	Injection site reactions, Rash	70% (√)	Positive	71–100	2.5	1.8
Simulect^®^ (Basiliximab)	IL-2	Saturates IL-2 receptors, prevents T cell + B cell activation	Immunogenicity, hypersensitivity, Rash	Uncommon 1/1000	Fever, acne. Rash, skin ulceration	54% (X)	Positive	31–70	2.0	1.1
Mabthera^®^ (Rituximab)	B cell	Destroys normal & malignant CD20+ B cells	Fatal IRs, Hypersensitivity, anaphylaxis, Cutaneous SJS, TEN	Common (10%)	Fever, urticaria/rash	50% (√)	Positive	31–70	2.0	1.0
Arzerra^®^ (Ofatumumab)	CD-20	Inhibits early B cell activation	Cutaneous: Rash, urticaria,	Common (10%)	Rash, pyrexia	50% (√)	Positive	31–70	1.7	0.9
Erbitux^®^ (Cetuximab)	EGFR	Targets EGFR expressing cells	Serious IR, dermatologic toxicity, rash, xeroderma, inflammation	Common (10%)	Urticaria, Rash, Pyrexia	50% (√)	Positive	31–70	1.7	0.8
Avastin^®^ (Bevacizumab)	VEGF	Binds to and inhibits VEGF	Hypersensitivity and infusion reactions: Rash, urticaria.	Common (8.4%)	Dry skin, exfoliative dermatitis	50% (√)	Positive	31–70	N/A	N/A
Inflectra^®^ (Infliximab)	TNFα	Neutralizing bind to TNFα	Anaphylaxis, IRs, vasculitis; SJS; EM; psoriasis	Common (3%)	Fever, chills, Anaphalaxis	40% (√)	Positive	31–70	1.6	0.6
Simponi^®^ (Golimumab)	TNFα	Targets soluble and transmembrane TNFα	Skin exfoliation, rash	Common (2%)	Injection site reactions, erythema, urticaria, induration, Psoriasis, Palmar/Plantar	40% (√)	Positive	31–70	1.7	0.7
Remicade^®^ (Infliximab)	TNFα	Neutralizing bind to TNFα	Anaphylaxis, IRs, vasculitis, SJS; EM; psoriasis	Common (3%)	Urticaria, anaphylaxis	35% (√)	Positive	31–70	1.5	0.6
					erythematous rash					
Vectibix^®^ (Panitumumab)	EGFR	Blocks signals on EGFR expressing cancer cells	Dermatologic toxicity, IR	Uncommon <1%	Severe skin toxicity (16%)	30% (√)	Weak Positive	10–30	1.3	0.4
Cimzia^®^ (Certolizumab)	TNFα	Binds free TNFα preventing its action	Allergic reactions, Dermatitis	Common (1 to 2.5%)	Pyrexia, Rash (0.3%)	30% (X)	Weak Positive	10–30	1.4	0.4
Tysabri^®^ (Natalizumab)	α4-integrin	Prevents immune cells crossing blood vessel walls	Hypersensitivity, Rash	Uncommon <1%	Hypersensitivity, anaphylaxis	20% (√)	Weak positive	10–30	1.6	0.3
Enbrel^®^ (Etanercept)	TNFα	Binds TNF prevents its binding to WBCs	Psoriasis, allergic reactions, autoimmune reactions	Common (4%)	Rash, Pyrexia	10% (X)	Weak positive	10–30	1.8	0.2

## Data Availability

The original data presented in the study are included in the article; further inquiries can be directed to the corresponding author.
